# Chronic arsenic toxicity

**DOI:** 10.1093/omcr/omad110

**Published:** 2023-10-23

**Authors:** Akshay Kor, Lokendra Singh Thakur, Kamalesh Tayade

**Affiliations:** Department of Cardiology, LTMMC and GH, Mumbai, India; Department of Cardiology, LTMMC and GH, Mumbai, India; Department of Neurology, LTMMC and GH, Mumbai, India

A 18-year-old man presented to the medicine casualty with palpitations and chest discomfort for 1 hour. An electrocardiogram was performed which showed supraventricular tachycardia (Fig. 1) with heart rate of 180 beats per minute, which reverted to sinus rhythm with administration of an intravenous beta blocker. The echocardiogram of his heart did not reveal any structural or functional heart abnormality. His serum electrolytes were normal. Throughout the patient’s hospital course he reported tingling and numbness involving both hands and feet for the last six months which was progressive in nature. Further questioning revealed consumption of homeopathic medications for the last 2 years as a health supplement. On further investigation he was found to have sensory motor axonal neuropathy involving all four limbs. On examination of the patient’s hand and back, he was found to have diffuse hyperpigmented spots which were brown in color giving characteristic ‘rain drop’ appearance (Figs 2–4).

**Figure 1:**
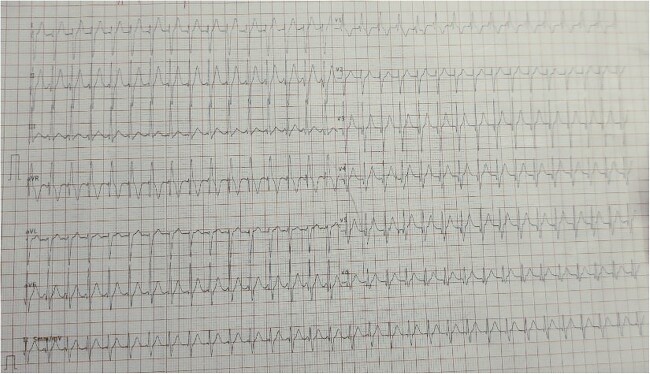
EKG showing supraventricular tachycardia.

**Figure 2:**
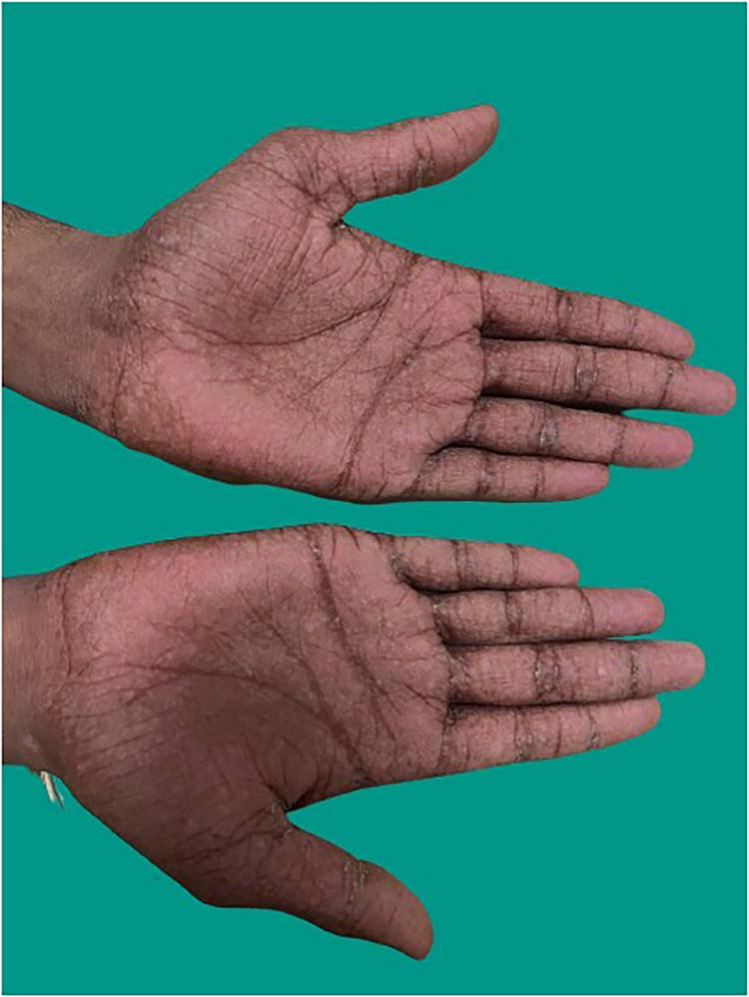
Rain drop hyperpigmentation in both hands.

**Figure 3:**
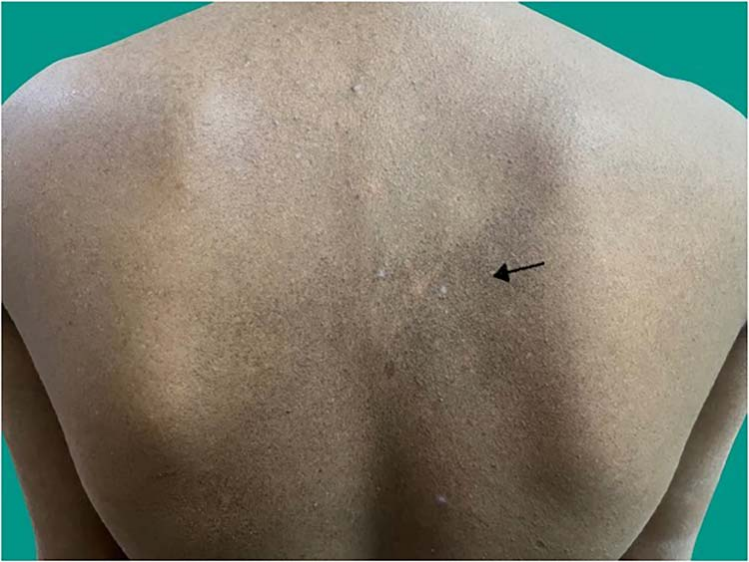
Hyperpigmentation in upper back region.

**Figure 4:**
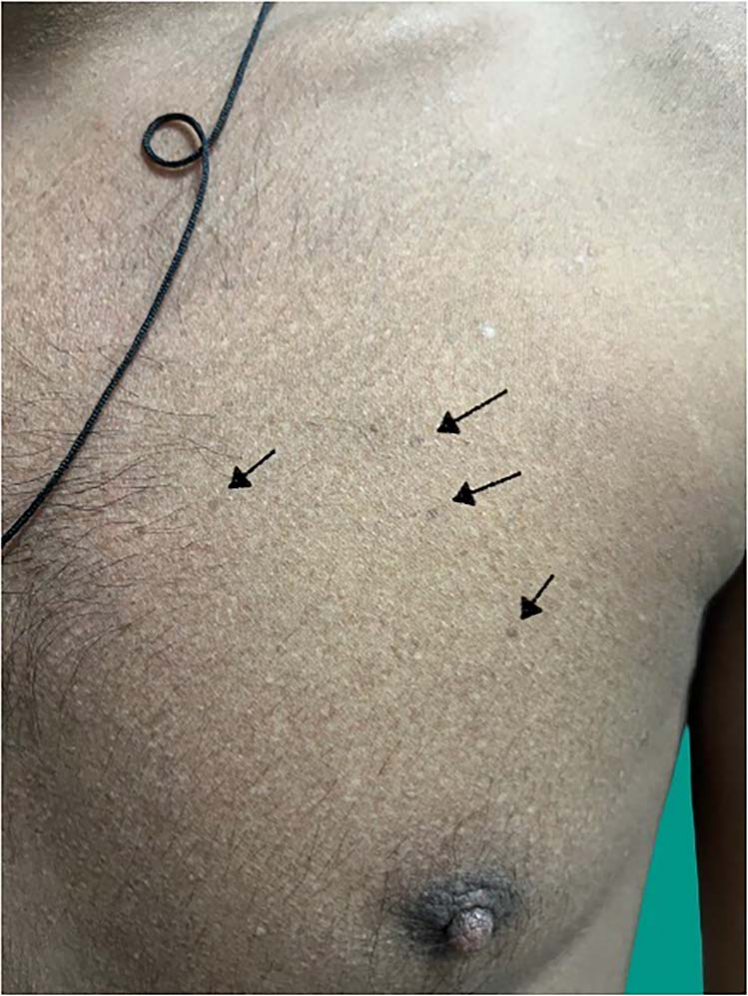
Hyperpigmentation in upper chest region.

Laboratory studies showed blood arsenic level of 4767 mcg/dl (reference <35 mcg/dl). A diagnosis of chronic arsenic toxicity with cardiac, dermatological and neurological manifestations was made. The homeopathic medications were discontinued. At a 6-month follow-up visit his skin changes were resolved and neurological symptoms were partially improved. He did not have further episodes of palpitations, and his beta blockers were tapered and discontinued (Table 1).

**Table 1:** Homeopathic and ayurvedic medications and associated identified heavy metals

	Homeopathic/Ayurvedic medication	Heavy metal identified	Reference
1.	Balsamodedron mukul	Lead, Mercury, Arsenic	[1]
2.	Arsenicum Sulfuratum	Arsenic	[2]
3.	Arsenic Bromide	Arsenic	[2]
4.	Mercurius 6a	Mercury	[3]
5.	Bryonia n 200	Lead, Chromium	[4]
6.	Allium Cepa-200	Lead, Copper, Chromium	[4]
7.	Schisandra chinensis	Copper	[5]
8.	*Plantago asiatica* L	Arsenic	[5]
9.	*Curcuma longa* L	Cadmium	[5]
10.	*Chrysanthemum indicum* L.	Mercury	[5]
11.	*Tetradium ruticarpum*	Lead	[5]

## CONFLICT OF INTEREST STATEMENT

None declared.

## FUNDING

No funding received.

## PATIENT CONSENT

Obtained.
